# Unmasking cor triatriatum dexter in adult patients with atrial arrhythmia

**DOI:** 10.21542/gcsp.2026.23

**Published:** 2026-06-30

**Authors:** Abdul Hakim Almakadma, Diana Inshyna, Parkha Khan, Mohamed Sabra, Ramzi Ibrahim, Meena Farid, Umida Burkhanova, Davendra Mehta, Farzane Saeidifard, Hal L. Chadow

**Affiliations:** 1 Department of Medicine, Division of Cardiovascular Diseases, Brookdale University Hospital & Medical Center, Brooklyn, New York, USA; 2 Department of Medicine, Brookdale University Hospital & Medical Center, Brooklyn, New York, USA; 3 Department of Cardiovascular Medicine, Mayo Clinic, Phoenix, Arizona, USA; 4 Department of Cardiovascular Medicine, SUNY Downstate, Brooklyn, New York, USA; 5 Al-Sabah Arrhythmia Institute, Mount Sinai Morningside Hospital, Icahn School of Medicine at Mount Sinai, New York, New York, USA

## Abstract

Cor triatriatum dexter is a rare congenital cardiac anomaly characterized by persistence of a membranous structure that divides the right atrium into two chambers. Although often asymptomatic, cor triatriatum dexter may present in adulthood with nonspecific symptoms and atrial arrhythmias, including atrial fibrillation and atrial flutter. We present a case series of four adult patients in whom cor triatriatum dexter was incidentally diagnosed during cardiac evaluation for diverse clinical presentations. Patients ranged in age from 51 to 75 years and presented with new-onset seizures, ischemic stroke evaluation, heart failure exacerbation, and recurrent atrial arrhythmias. In all cases, diagnosis was established using echocardiographic imaging, with transesophageal echocardiography providing definitive visualization when transthoracic studies were nondiagnostic. This series highlights the diagnostic challenges of cor triatriatum dexter in adults and suggests that the condition may be underrecognized. Furthermore, altered right atrial anatomy may be associated with atrial arrhythmia maintenance through structural and conduction alterations of the right atrium.

## Introduction

Cor triatriatum dexter (CTD) is a rare congenital anomaly characterized by the division of the right atrium (RA) into two chambers by a membrane, resulting from the persistence of the right valve of the embryonic sinus venosus. The incidence of CTD is estimated to be about 0.1% of congenital cardiac malformations^1^. Diagnosis of CTD in adulthood is uncommon and often incidental. Many cases are identified through imaging studies such as echocardiography or computed tomography during evaluations for other cardiac conditions. The literature includes reports of adult patients diagnosed with CTD at various ages, often during investigations for nonspecific symptoms or other cardiac anomalies^1,2^. CTD has been suggested to alter right atrial anatomy and conduction pathways. Its presence may be associated with arrhythmia initiation or persistence, particularly when the membrane involves critical areas like the cavo-tricuspid isthmus^3^. In this report, we present four cases of CTD that were incidentally diagnosed during the work up for their primary presentation.

## Case presentations

### Case #1

A 61-year-old male with medical history of hypertension, chronic kidney disease, insulin-dependent type 2 diabetes mellitus, heart failure with improved ejection fraction from 45–50% to 55%, atrial fibrillation (AF), who presented with new-onset seizures, uncontrolled hyperglycemia 658 mg/dL (normal range: 70–99 mg/dl), and impaired consciousness. On admission, he was febrile (38.3 ° C) and tachycardic with a heart rate of 152. An electrocardiogram was performed and revealed AF with rapid ventricular response. Chest radiography showed no pulmonary edema. Physical exam revealed no murmurs or signs of heart failure. Patient was intubated for airway protection and started on anti-epileptics and empirical antibiotic therapy.

**Figure 1.**
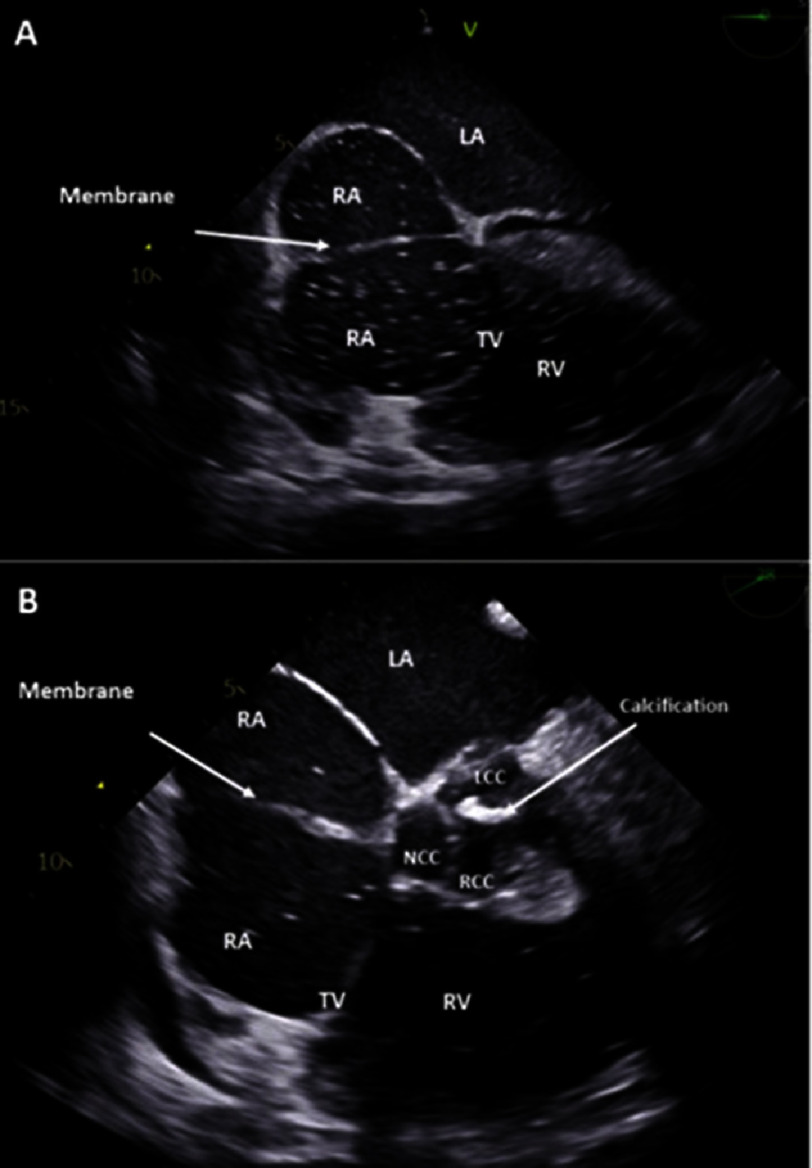
Transesophageal echocardiogram of patient 1. (A) Mid-esophageal view illustrating the right atrium divided by a membrane. (B) Reveals the right atrium membrane at the level of the aortic valve with evidence of calcification on the left coronary cusp. LA, Left Atrium; RA, Right Atrium; TV, Tricuspid Valve; RV, Right Ventricle; NCC, Non-coronary Cusp; LCC, Left Coronary Cusp; RCC, Right Coronary Cusp.

Transthoracic echocardiography (TTE) revealed aortic valve thickening and calcification, predominantly involving the left coronary cusp, the RA was not well visualized. To exclude infective endocarditis, a transesophageal echocardiogram (TEE) was performed, identifying a possible fenestrated membrane in the RA with no significant obstruction, consistent with CTD (Figure 1; Video S1). Patient was extubated on day four of admission, lumbar puncture and serial blood cultures were negative and antibiotics were stopped after 6 days. Magnetic resonance imaging of the brain revealed 2.9 cm dumbbell-shaped cavity in the posterior frontal lobe. Patient was discharged on Apixaban, antiepileptics and plan for outpatient follow up with neurology.

### Case #2

A 69-year-old male with a medical history of hypertension, type 2 diabetes mellitus, heart failure with improved ejection fraction (from 35–40% to 55%), and paroxysmal AF and atrial flutter (AFl) presented with palpitations and associated hemodynamic instability. This required synchronized electrical cardioversion with 50 joules, which successfully restored normal sinus rhythm (NSR) on electrocardiogram. He was then discharged with a plan for an outpatient electrophysiology study and catheter ablation.

**Figure 2.**
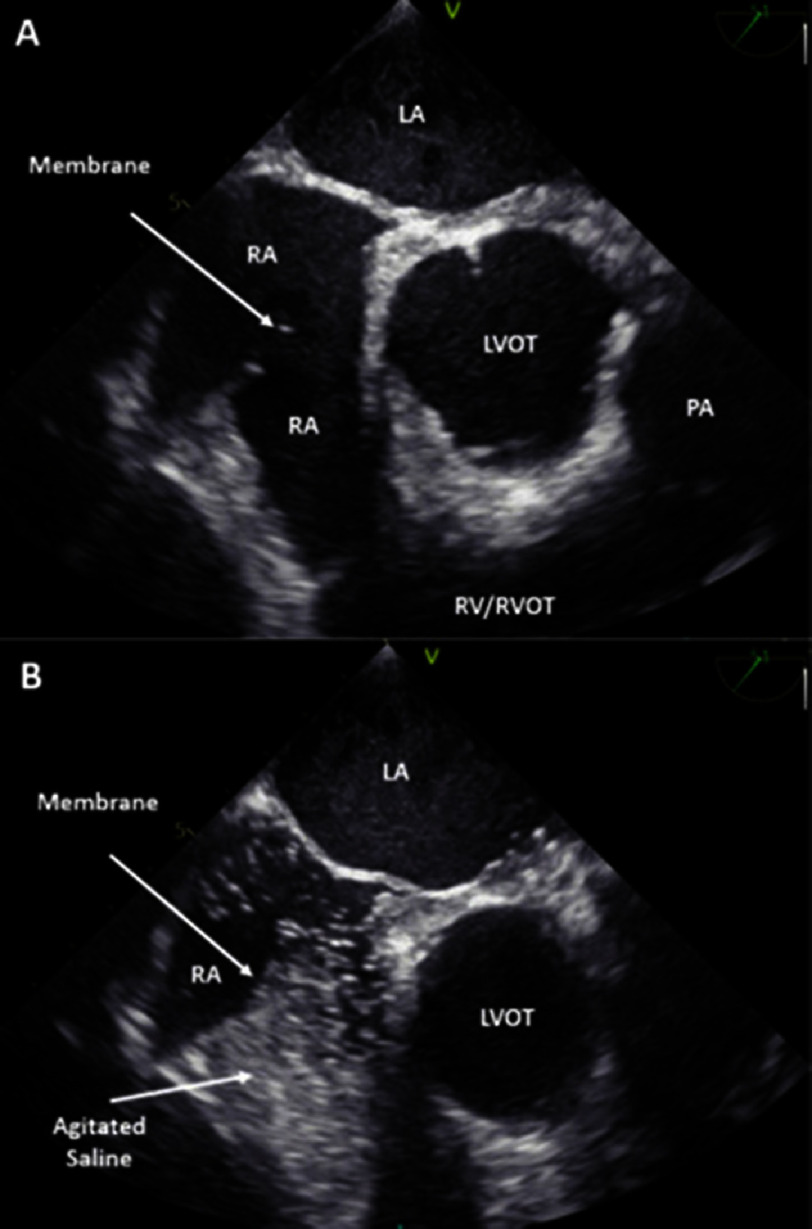
Transesophageal echocardiogram of patient 2. (A) Mid-esophageal view illustrating the membrane dividing the right atrium pre-agitated saline and (B) post agitated saline. LA, Left Atrium; RA, Right Atrium; TV, Tricuspid Valve; RV/RVOT, Right Ventricle/Right Ventricular Outflow Tract; LVOT, Left Ventricular Outflow Tract; PA, Pulmonary Artery.

A few days following discharge, the patient returned with recurrent palpitations that transiently resolved with a single dose of ibutilide. He was readmitted due to recurrent episodes of palpitations. He was hemodynamically stable with a heart rate of 144 bpm, and electrocardiogram revealed atrial flutter (AFl) with a 2:1 conduction pattern. He received metoprolol and diltiazem and was started on an amiodarone infusion, with subsequent conversion to NSR, and was scheduled for an inpatient electrophysiology study and catheter ablation. TEE performed to rule out left atrial thrombus prior to the procedure revealed a normal left ventricular ejection fraction and a moderately dilated right atrium with a fenestrated membrane in the middle, without obstruction, consistent with CTD (Figure 2; Video S2), as well as a moderate-to-large patent foramen ovale (PFO). The patient subsequently underwent successful cavotricuspid isthmus radiofrequency ablation with no complications and was discharged with a follow-up appointment in the heart failure clinic. Over a 6-month follow-up period, he remained in NSR with no further readmissions and was referred for possible PFO closure.

### Case #3

**Figure 3.**
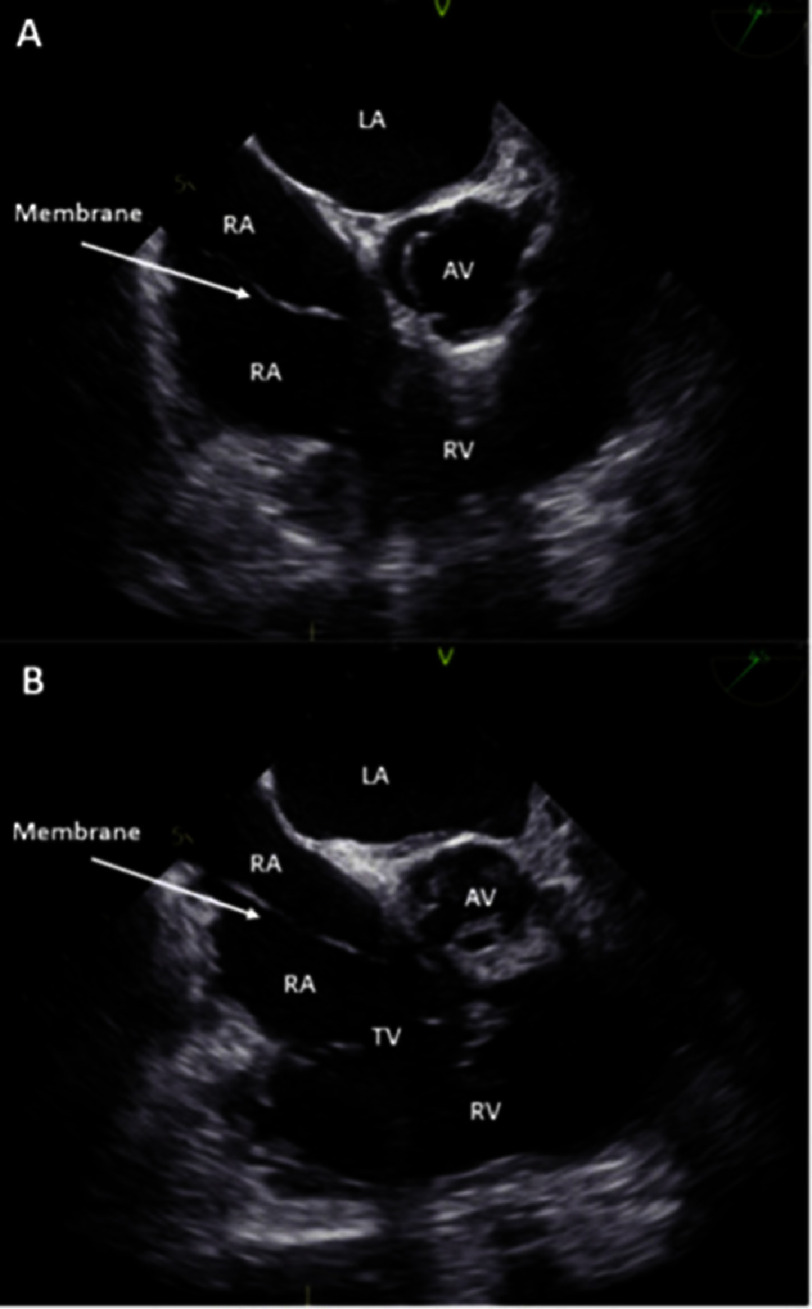
Transesophageal echocardiogram of patient 3. Mid-esophageal view illustrating the membrane dividing the right atrium. LA, Left Atrium; RA, Right Atrium; TV, Tricuspid Valve; RV, Right Ventricle; AV, Aortic Valve.

A 51-year-old female with a medical history of hypertension, hypothyroidism, and type 2 diabetes mellitus presented with aphasia and agraphia. MRI confirmed an acute left cortical ischemic infarct with prior chronic occipital and cerebellar infarcts. TTE was unremarkable; however, due to concern for a cardioembolic source of her strokes, a TEE was performed. This ruled out a PFO after the injection of agitated saline but revealed a mildly dilated right atrium with a fenestrated membrane, without obstruction, with one end connected to the right ventricular septum beneath the tricuspid leaflet and the other extending to the inferior vena cava, suggestive of CTD (Figure 3; Video S3).

No thrombus was visualized; however, a mobile echo-density measuring 0.3 × 0.8 cm was noted on the tricuspid valve, suggestive of a vegetation. Blood cultures were collected and showed no growth at 72 h. The patient did not exhibit any signs of systemic infection and therefore did not require antibiotic treatment. Inpatient telemetry monitoring for 48 h revealed no arrhythmic events; therefore, an implantable loop recorder (ILR) was placed for prolonged cardiac monitoring, and the patient was discharged with a plan for follow-up. The patient was followed as an outpatient by cardiology, remained asymptomatic, and ILR monitoring revealed no events over the next 6 months.

### Case #4

A 75-year-old female with hypertension, hyperlipidemia, insulin-dependent type 2 diabetes mellitus, and triple-vessel coronary artery disease with prior coronary artery bypass grafting was referred to the hospital from her primary care clinic due to hyperkalemia (K^+^ 6.5 mEq/L; reference range 3.5–5.1 mEq/L).

She reported shortness of breath on exertion. TTE revealed new-onset heart failure with moderately reduced ejection fraction (40–45%), a markedly dilated left atrium, and a dilated right atrium. She was discharged with a follow-up appointment in the heart failure clinic and a referral for cardiac rehabilitation. At her 2-week follow-up appointment, she was diagnosed with new-onset AF and was started on apixaban.

**Figure 4.**
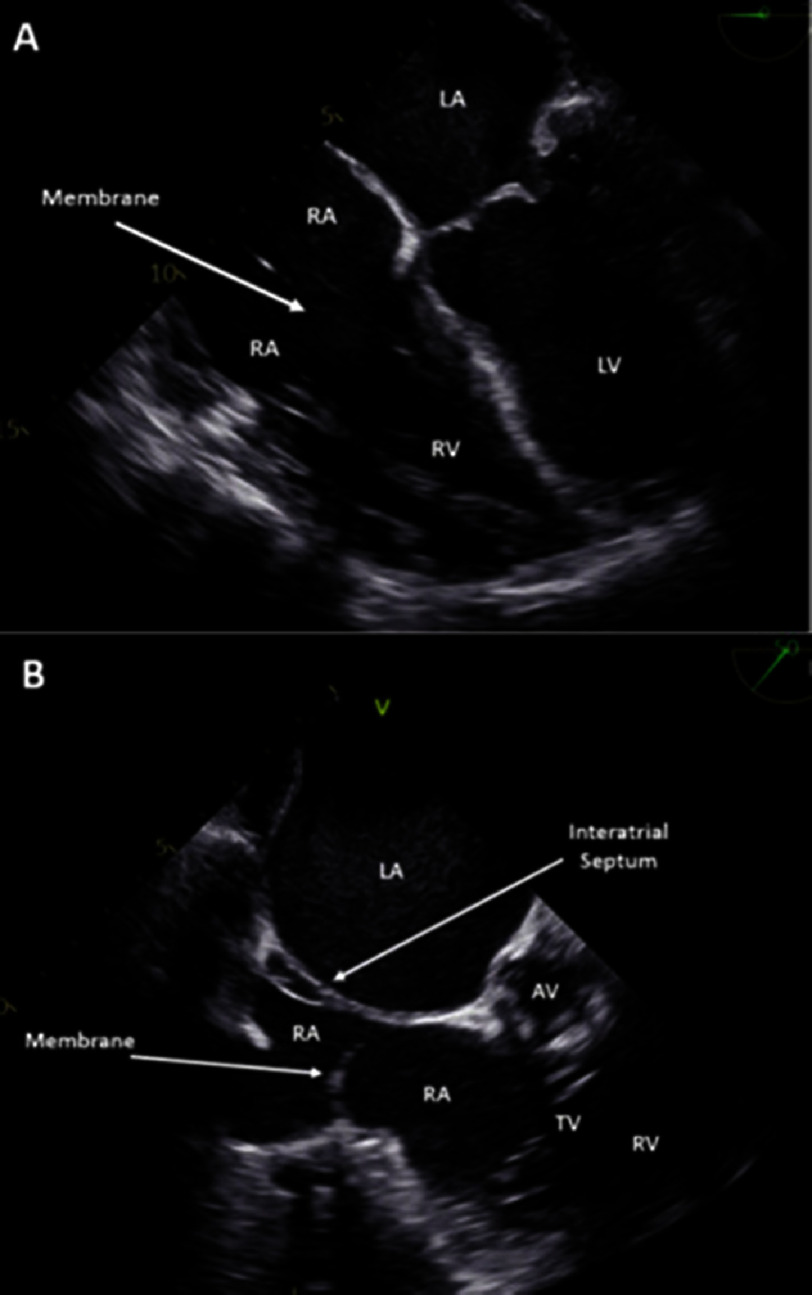
Transesophageal echocardiogram of patient 4. (A) Transesophageal echocardiogram with mid-esophageal four chamber view illustrating the right atrium membrane. (B) Off-axis view to better visualize right atrium membrane. LA, Left Atrium; RA, Right Atrium; TV, Tricuspid Valve; RV, Right Ventricle; LV, Left Ventricle; AV, Aortic Valve.

A month later, she presented with worsening shortness of breath and lower extremity swelling. She was admitted for a heart failure exacerbation and was started on diuretics. After optimization of her volume status, she was scheduled for a TEE with cardioversion for her AF. TEE was significant for heart failure with reduced ejection fraction (30–35%) and a thrombus at the junction of the left atrium and left atrial appendage (LAA), as well as a moderately dilated right atrium with a fenestrated membrane without obstruction, consistent with CTD (Figure 4; Video S4). Cardioversion was deferred due to the LAA thrombus. She was discharged with a wearable cardioverter-defibrillator. The patient was followed by electrophysiology for 6 months; repeat TEE showed a persistent LAA thrombus and an ejection fraction of 25–30%. Ablation was deferred, and the patient was scheduled for an implantable cardioverter-defibrillator.

## Discussion

In this case series, CTD was incidentally identified in four adult patients (Table 1) undergoing TEE for evaluation of arrhythmias, embolic stroke, or structural heart disease. Although CTD is a rare congenital anomaly, its presence in our patients with electrical abnormalities suggests a possible association with their underlying clinical presentations.

**Table 1:** Patient demographics, comorbidities, CTD anatomy, atrial structure and function, and AF characteristics.

	**Patient 1**	**Patient 2**	**Patient 3**	**Patient 4**
**Patient Profile**				
**Age (y)**	61	69	51	75
**Sex**	Male	Male	Female	Female
**Ethnicity**	Black/African American	Black/African American	Black/African American	Black/African American
**BMI (kg/m^2^)**	36.18	36.7	36.2	26.2
**NYHA class**	NYHA Class I	NYHA Class II	NYHA Class I	NYHA Class II
**Prior congenital heart disease or surgery**	No	No	No	No
**CTD Anatomy**				
**Imaging modality**	TEE	TEE	TEE	TEE
**Attachment sites**	Middle RA	Middle RA	One end of the membrane might be connected to the right ventricular septum under the tricuspid leaflet. The other end extends into the inferior vena cava	RA
**Fenestration status**	Fenestrated membrane	Fenestrated membrane	Fenestrated membrane	Fenestrated membrane
**Obstruction**	No significant obstruction	No	No	No
**RA area/volume, TR Grade, estimated PASP.**	RA severely dilated, no tricuspid regurgitation	Mild RA dilation, Trace tricuspid regurgitation	RA mildly dilated, moderate tricuspid regurgitation. 0.8 × 0.3 mobile echodensity on the septal leaflet suggestive of vegetation.	RA moderately dilated, mild to moderate tricuspid regurgitation
**Functional and Structural Findings**				
**LVEF %**	50–55%	55–60%	55–60%	30–35%
**Diastolic function**	N/A	Grade 2	Grade 1	N/A
**LAA thrombus**	No	No	No	Yes
**PFO present?**	No	Yes	No	No
**Atrial Fibrillation Characteristics**				
**Presence of AF**	Paroxysmal	Paroxysmal	N/A	Yes
**Diagnostic timing**	AF preceded CTD	AF preceded CTD	N/A	AF preceded CTD
**Documentation modality**	Electrocardiogram	Electrocardiogram	Electrocardiogram, Holter, ILR	Electrocardiogram
**Co-arrhythmias**	AF	Atypical AFl	N/A	AF

**Notes.**

AF Atrial Fibrillation BMI Body Mass Index NYHA New York Heart Association TEE Transesophageal Echocardiography RA Right Atrium IVC Inferior Vena Cava TR Tricuspid Regurgitation LA Left Atrium LVEF Left Ventricular Ejection Fraction LAA Left Atrial Appendage PFO Patent Foramen Ovale CTD Cor Triatriatum Dexter EKG Electrocardiogram ILR Implantable Loop recorder AFl Atrial Flutter.vv

CTD is often found incidentally and is not associated with long-term complications or an increased risk of developing comorbidities in the absence of membrane-related obstruction or associated congenital cardiac anomalies. However, it has been suggested that the abnormal septation in CTD may alter intracardiac flow patterns within the RA, although direct hemodynamic consequences in non-obstructive variants remain incompletely characterized^3,4^.

In our series, atrial arrhythmias preceded the diagnosis of CTD in three of four patients, and the remaining patient had no documented arrhythmia. This temporal relationship limits any inference of causality with respect to arrhythmia initiation. Accordingly, CTD in this cohort should be interpreted as an associated structural finding rather than a primary trigger of atrial arrhythmia.

However, the absence of a temporal relationship does not exclude a contributory role in arrhythmia maintenance. AF and flutter are sustained not only by triggers but also by an underlying substrate that permits reentry and conduction heterogeneity. The presence of a right atrial membrane in CTD may introduce localized conduction barriers and modify critical regions such as the cavotricuspid isthmus. These changes may facilitate the persistence or recurrence of atrial arrhythmias, even if they are not the initiating factor^5,6^. CTD has also been recognized in the literature as being associated with congenital anomalies, including PFOs, as in our second patient^1,7^.

Although the reported incidence is estimated at approximately 0.1% of congenital cardiac malformations, our incidental findings raise the possibility that CTD may be underreported. The true incidence may be higher and remains difficult to determine, particularly in nonobstructive variants, as clinical suspicion does not arise unless other cardiac defects or symptoms are present. All four cases in our series were identified incidentally during TEE performed for other clinical indications. Although these findings arose from a limited number of examinations at a single community hospital, they suggest that CTD may go unrecognized on transthoracic imaging and become apparent only with more detailed transesophageal evaluation.

CTD is most often diagnosed using TTE and TEE which enable detailed assessment of associated cardiac anomalies, accurate measurement of the defect, and evaluation of left ventricular ejection fraction. When further anatomical clarification is needed to guide clinical decision-making, cardiac CT and MRI may also be used^8^.

The management of CTD in patients presenting with AF or AFl involves addressing both the structural cardiac anomaly and the arrhythmic condition. Treatment strategies are tailored based on symptom severity, hemodynamic impact, and the presence of associated cardiac anomalies. Although medical management with rate and rhythm control medications can be considered, it is often a temporizing measure, as it does not address the underlying structural defect.

Surgical resection of the CTD membrane is the most effective intervention in patients with significant obstruction to right atrial inflow or an associated right-to-left shunt^3^.

Our second patient experienced recurrent palpitations requiring multiple readmissions for symptomatic AFl not controlled with oral rate- and rhythm-control agents. In patients in whom AF/AFl is the predominant symptom with absent or insignificant obstruction, radiofrequency catheter ablation is the treatment of choice for rhythm control^9^.

There are certain limitations to effective rhythm-control strategies, ranging from something as simple as loss to follow-up, as with our first patient, to contraindications such as the presence of a left atrial thrombus, as in our fourth patient. Moreover, the presence of an atrial membrane may pose a challenge during procedures such as ablation. The membrane can not only complicate mapping by creating anatomical barriers but also hinder catheter advancement^10^. An individualized, case-by-case approach is required to ensure favorable outcomes.

## What we have learned?

• CTD may be underrecognized in adults and TTE often fails to identify right atrial membranes.
• In this series, CTD was an associated structural finding rather than a causal trigger of atrial arrhythmia.
• Recognition of CTD is clinically relevant, particularly prior to electrophysiologic interventions, as right atrial membranes may influence mapping, catheter manipulation, and procedural strategy.

### CRediT author statement

**Conceptualization:** Abdul Hakim Almakadma, Farzane Saeidifard

**Data curation:** Abdul Hakim Almakadma, Diana Inshyna, Parkha Khan

**Writing–Original Draft Preparation:** Abdul Hakim Almakadma, Diana Inshyna, Parkha Khan, Mohamed Sabra

**Writing–Review & Editing:** Abdul Hakim Almakadma, Ramzi Ibrahim, Meena Farid, Umida Burkhanova, Farzane Saeidifard, Davendra Mehta, Hal L. Chadow

## References

[1] Simsek Z, Koza Y, Tas H (2014). Cor triatriatum dexter, atrial septal defects, and pulmonary stenosis—a rare association. Echocardiography.

[2] Low TT, Uy CCC, Wong RCC (2014). Unique sail-like structure of cor triatriatum dexter in three-dimensional echocardiogram. Echocardiography.

[3] Nageh MF, Watanabe CT, Chou ET (2013). Ablation of isthmus and non-isthmus-dependent flutters in a patient with cor triatriatum dexter. Europace.

[4] Fuentes Rojas SC, Lawrie G, Faza NN (2022). Cor triatriatum dexter: An innocent bystander. Methodist DeBakey Cardiovascular Journal.

[5] Bernier M, Marelli AJ, Pilote L, Bouchardy J, Bottega N, Martucci G, Therrien J (2010). Atrial arrhythmias in adult patients with right- versus left-sided congenital heart disease anomalies. The American Journal of Cardiology.

[6] Rodriguez Ziccardi M, Goyal A, Maani CV (2025). Atrial flutter. StatPearls.

[7] Sahin T, Bildirici U, Kandemir C, Celikyurt U, Ural D, Komsuoglu B (2008). Infective endocarditis in the setting of infundibular–valvular pulmonary stenosis with incomplete cor triatriatum dextrum and patent foramen ovale. International Journal of Cardiology.

[8] Meel R, Sothoane KL, Variava E (2025). An incidental finding of Cor Triatriatum in an adult with left ventricular dysfunction and atrial flutter. Oxford Medical Case Reports.

[9] Page RL, Joglar JA, Caldwell MA, Calkins H, Conti JB, Deal BJ, Estes NAM, Field ME, Goldberger ZD,  Hammill SC, Indik JH, Lindsay BD, Olshansky B, Russo AM, Shen W-K, Tracy CM, Al-Khatib SM (2016). 2015 ACC/AHA/HRS guideline for the management of adult patients with supraventricular Tachycardia: A report of the american college of cardiology/american heart association task force on clinical practice guidelines and the heart rhythm society. Circulation.

[10] Karimianpour A, Cai AW, Cuoco FA, Sturdivant JL, Litwin SE, Wharton JM (2021). Catheter ablation of atrial fibrillation in patients with cor triatriatum sinister; case series and review of literature. Pacing and Clinical Electrophysiology: PACE.

